# Electrochemical corrosion protection of neat and zinc phosphate modified epoxy coating: A comparative physical aging study on Al alloy 6101

**DOI:** 10.3389/fchem.2023.1142050

**Published:** 2023-02-14

**Authors:** Ahsan Riaz Khan, Hai-Jun Zhang, Zhang Jun, Zheng Maosheng, Sayed M. Eldin, Imran Siddique

**Affiliations:** ^1^ Department of Interventional and Vascular Surgery, Shanghai Tenth People’s Hospital, Tongji University School of Medicine, Shanghai, China; ^2^ National United Engineering Laboratory for Biomedical Material Modification, Branden Industrial Park, Qihe Economic and Development Zone, Dezhou City, Shandong, China; ^3^ Department of Chemical Engineering, Northwest University, Xi’an, China; ^4^ Research Center for Translational Medicine, Shanghai East Hospital, School of Medicine, Tongji University, Shanghai, China; ^5^ Shanghai Institute of Stem Cell Research and Clinical Translation, Shanghai, China; ^6^ Center of Research, Faculty of Engineering, Future University in Egypt, New Cairo, Egypt; ^7^ Department of Mathematics, University of Management and Technology, Lahore, Pakistan

**Keywords:** modified epoxy, zinc phosphate, physical aging, electrochemical analysis, corrosion resistance

## Abstract

Optimizing the pigment volume concentration of zinc phosphate pigments can protect Al alloy 6101 from alkaline media. Additionally, zinc phosphate pigments form a shielding film on the substrate and facilitate stopping the penetration of aggressive corrosion ions. The efficiency of eco-friendly zinc phosphate pigments is almost 98% during the corrosion analysis. A comparative study of physical aging of neat epoxy and Zinc Phosphate (ZP) pigment-modified epoxy coatings on Al alloy 6101 was conducted in Xi’an, China, for one year in all four seasons, where in summer for 3 months, results degraded more due to high UV radiation and humidity; it is found that peeling force of ZP pigments modified epoxy coatings is 50% higher of than that of the neat epoxy coatings though both peel-off adhesion strength and scratch test visibility decreased in both coatings; The electrochemical resistance of ZP pigments modified epoxy coatings is about 30% higher of than that of neat epoxy coatings, the corrosion rate of ZP pigments modified epoxy coatings is about 70% lower of than that of neat epoxy coatings, moreover the gloss retention is 20% higher in the modified epoxy; Optical surface observation of the coatings showed that the ZP modified epoxy coating could effectively restrict the crack and shrinkage in coatings after aging experimentation in the natural environment.

## Graphical Abstract

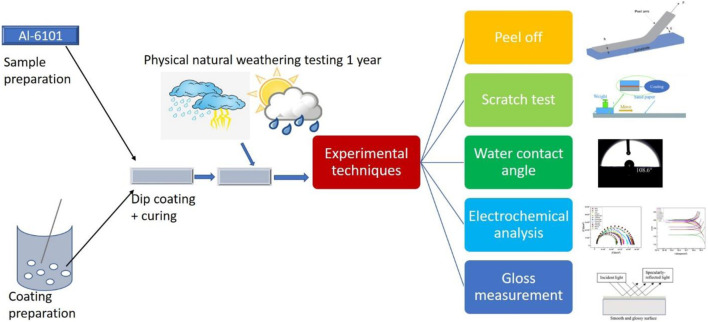

## 1 Introduction

Epoxy resins are thermosetting polymers frequently used in electrical appliances, paints, varnishes, and adhesives (Jin et al., 2015). Epoxy is also frequently utilized as a matrix material in fiber composites in the aerospace and wind energy industries because of its high specific stiffness, specific strength, electrical insulating qualities, resistance to corrosion, chemical compatibility with reinforcing fibers, and relative ease of production (Jin et al., 2015; Khan et al., 2022a). Epoxies are ideal for such applications. In many of these applications, epoxies and epoxy composites are frequently exposed to long-term sustained levels of high temperature, dampness, electric fields, and other hostile conditions (Abdelkader and White, 2002; An et al., 2021). Epoxy resin ages due to prolonged exposure, which may decline its overall thermomechanical properties (Bradshaw and Brinson, 1997; Bell et al., 2021).

Usually, mechanical and chemical barrier properties of the epoxy coatings degraded due to the water adsorption (Bradshaw and Brinson, 1997; Calvez et al., 2021), the change in the chemical structure of the organic coatings is altered with the formation of hydrogen bonding through water uptake, especially in epoxies that leads towards the formation of the conductive paths for ions (Da Silva et al., 1999; Chen et al., 2022). Therefore, this process is the main cause of epoxy coatings’ degradation by water adsorption and breaking hydrogen bonding in the polymer structures (Da Silva et al., 1999; Chen et al., 2022; da Silva Lopes et al., 2020). Similarly, many other consequences due to water adsorption occur in the coating durability, like micro cracking, reduction of fracture toughness and loss of adhesion on the metal substrate, resulting in the disbandment of coatings (da Silva Lopes et al., 2020; Dang et al., 2014; Deyab et al., 2018).

Aluminum is the 13th element in the periodic table and the 3rd most chemical element after oxygen and silicon (de la Fuente, 2022). It is whitish and 8% of the Earth’s central mass. It was first discovered in 1824 and is widely used on a manufacturing scale (Fang et al., 2020; de la Fuente, 2022). Pure aluminum can be combined with other elements to enhance its properties, most notably, its strength. Metalloids and metallic elements were mixed to create an aluminum alloy (Fang et al., 2020; Vargel, 2020; de la Fuente, 2022). Wrought designation and cast designation systems are two ways to identify aluminum. The first digit (Xxxx) represented the primary alloying component added to the aluminum alloy and was used to determine the aluminum alloy series (1,000–8,000 series) (Lindsey, 1966). Alloys with atomic number 12 and semiconductors as primary alloying parts are versatile, heat treatable, have moderate strength, outstanding weldability, and smart corrosion resistance (Revie, 2008). Aluminum alloy 6101 is the most commonly used in the 6xxx metal (Al) alloy series. It produces windows, door elements, fuses, electrical conductors, and screw machine elements (Revie, 2008; Vargel, 2020).

The function of coatings protection is to prevent the substrate from corrosion, scratch, peel-off, etc., which mainly depends upon the chemical and mechanical properties of the coating and adhesive performance with the substrate (Grzmil et al., 2007; Hao et al., 2013). A significant change in the mechanical properties during service is undeniable due to humidity, temperature, UV rays, shrinkage and cracks appearance, swelling by water, and crosslink density (Huang et al., 2006; Jin et al., 2015). Most studies regarding protective properties and their degradations of coatings focused on UV absorption and irradiation. At the same time, the effects of humidity (moisture adsorption) and temperature changes could be very influential factors; comprehensive effects of many factors need to be paid more attention to especially (Järnström et al., 2008; Khan et al., 2022b).

Physical aging is known as structural relaxation, a kinetic phenomenon in which organic polymers relax when not in equilibrium because they are present at temperatures lower or higher than their glass transition temperature (Tg) (Perera, 2003; Le Guen-Geffroy et al., 2019). Additionally, previous studies have shown that the dominant change in the weathering process is the main reason for organic and polymeric material degradation. A possible reason for this coating degradation process could be the formulation, application, and curing of materials in many domains of the automobile, aerospace, and chemical industries (Lee and McKenna, 1988; Mayer et al., 2022).

In the last 30 years, many publications dedicated to the physical aging of polymeric materials, contrary to organic coatings, even of their huge significance, have not gained the same attention (Naderi and Attar, 2009; Oliveira et al., 2021). There are many reasons organic coatings have not been investigated, as they should be used in the coating and chemical industries. The main possible reason for this could be the complexity of the process, such as cross-linkage, leaching, loss of solvents, photo-oxidation, and degradation during the coating service life span (da Silva Lopes et al., 2020; Khan et al., 2022c). However, environmental conditions change drastically daily. These factors rapidly affect the physical aging of coatings; thus, the industry needs to prepare more effective and durable coatings (Le Guen-Geffroy et al., 2019).

**FIGURE 1:**
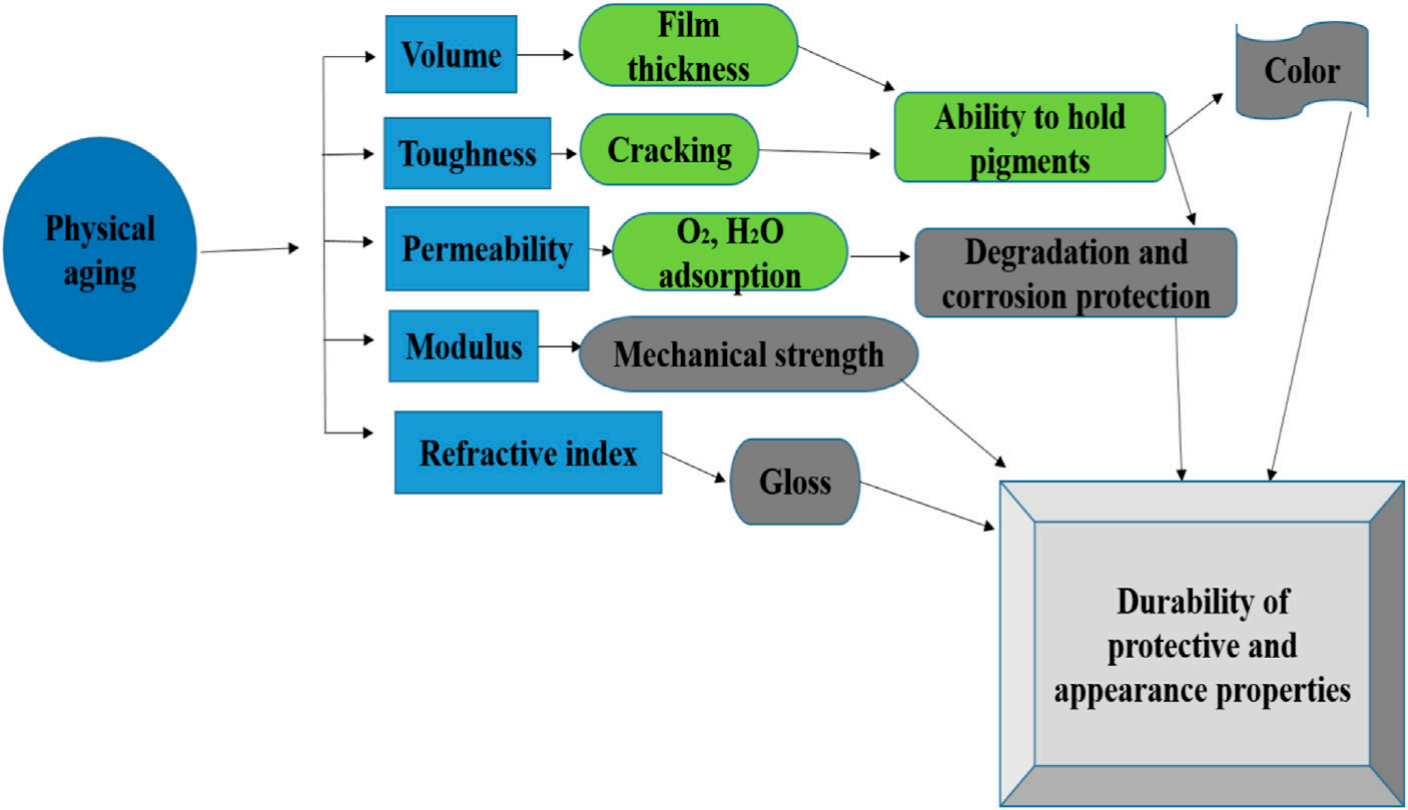
Potential influence of physical aging on the coating’s performance.

In general, many factors affect the durability and stability of the coatings, which include radiation, sunlight, pollutants, dust, oxygen, humidity, temperature, and wind speed. In most cases, the influential factors simultaneously affect coatings’ properties and accelerate mutually, such as natural exposure (Peng et al., 2016; Oliveira et al., 2021; Khan et al., 2022c). The potential Influence of physical aging on the coating’s performance is very rigorous in terms of resilience and durability during degradation see Figure 1 (Shi et al., 2008). Delimitations phenomenon and adhesive strength tests are sensitive to the weathering process of temperature and humidity changes, so the corresponding studies of the peel-off test, scratch test, and electrochemical test can be employed to characterize the degradation of comprehensive properties due to aging of natural exposure comparatively. Therefore, the effect of the addition of the inhibitive pigments on the anti-degradation of the pigment-modified epoxy coatings against natural exposure can be evaluated precisely (Shao et al., 2009; Shi et al., 2018).

This study elaborated on the efficiency of a neat epoxy coating modified with ZP pigments on Al alloy 6101, which was not previously explored. Moreover, physical aging under natural weather, including heavy/light rain (mechanical damage) during the whole year with a time of three months, including four seasons, while at high temperatures and humidity for both coatings, is being investigated by different experimental techniques in which the peel off test, scratch test, and electrochemical test, water contact angle test and gloss measurements are performed. Moreover, surface characterization is done by the scanning electron microscope and EDS analysis to confirm the distribution and elemental presence of the Zinc phosphate pigments. FTIR analysis was used to verify that no structural changes were observed after the epoxy modification with ZP pigments. The results showed that the protective efficiency of the modified coating with ZP pigments was comparatively higher than that of the neat epoxy.

## 2 Material and methods

### 2.1 Materials and coating preparation

As to the comparative study of the aging effects of protective properties of neat epoxy and ZP-modified epoxy coatings, both coatings on Al alloys are prepared by dipping methods. Al alloy 6101 used in this study is with compositions of Si: 0.3%–0.7%, Fe ≤ 0.50%, Cu ≤ 0.10%, Mn ≤ 0.03%, Mg: 0.35%–0.8% and Cr ≤ 0.03%.

The epoxy resin was brought from (Hubei Xinsihai Chemical Co., Ltd., China) and a solvent mixture of butyl acetate, ethylene glycol, and toluene with a mass ratio of 0.5: 0.5: 3. All these chemicals are with the purity of 99% from Wuxi Yasheng Chemical co., Ltd. The optimized ratio of epoxy to the solvent mixture is 1:4.5.

**FIGURE 2:**
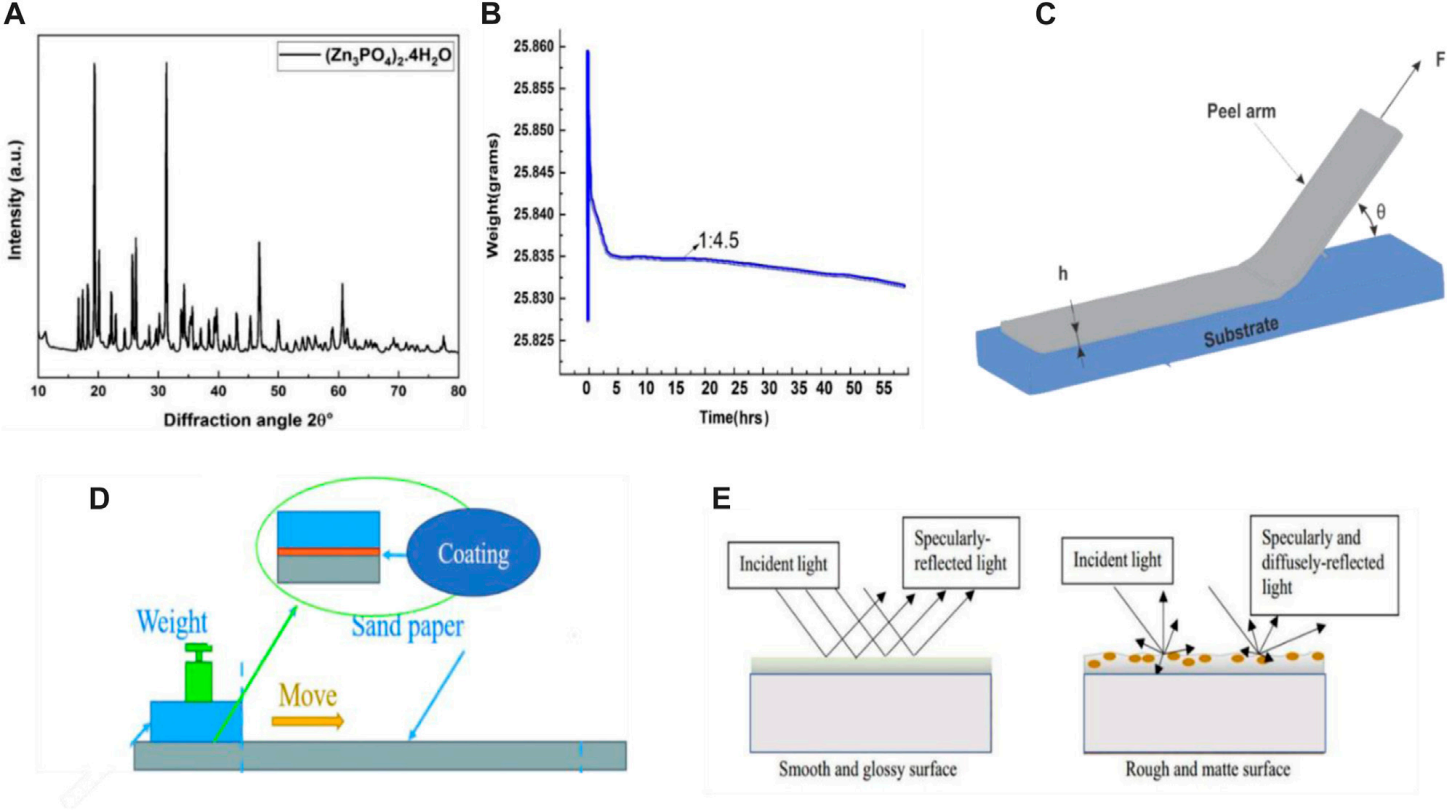
2**(A)** X-ray diffraction of ZP pigment, 2**(B)** typical evaporation process of coated sample with ratio of epoxy to solvent of 1:4.5, 2**(C)** peeling off model of a film with adhesion length *l* in contact with a substrate, 2**(D)** 5 schematic diagram of the scratch test and 2**(E)** schematic presentation mechanism of light interaction on the smooth and rough surface.

Subsequently, a high-speed sand mill was used to disperse the ZP pigments in epoxy coatings with varying concentrations of 0.5%, 0.6%, and 0.7% added to the neat epoxy resin solution. The size of samples of Al alloy 6101 is 40 × 20 × 5 mm, which is prepared by surface treatment grit-blasted and cleaned with acetone, acid, and base pre-treatment also done to substrate for better adhesion. ZP pigments were bought from (Sino Pharm Chemical Co., Ltd., China), and their technical information is listed in Table 1; their composition is already detailed and discussed in this research article (Zn_3_PO_4_)_2_.4H_2_O (Grzmil et al., 2007). Figure 2A Shows its XRD spectrum.

**TABLE 1:** Technical data of ZP pigments used for anti-corrosion coating.

Characteristics	Pigment density g/cm^3^	Particle average size µm	pH
Zinc phosphate (ZP)	3.2	2–4	7

### 2.2 Coating preparation method and procedure

Both neat epoxy resin and modified epoxy coating are prepared by dipping process according to the following procedure.

1. Immersing the Al alloy 6101 substrates into the solution (30 mL) for a half-hour;
2. Draw the substrate at a speed of 0.05 mm/s from the immersed solution to prepare a wet-coated sample;
3. The wet sample is dried at room temperature (about 24°C and 55% moisture) first; its weight change is recorded to evaluate the liquid evaporation rate of the epoxy coating every 2 min simultaneously; The typical evaporation process of the coated sample with the ratio of epoxy to a solvent mixture of 1:4.5 is shown in Figure 2B, which indicates that the evaporation rate is steady after four hours. No significant change in weight is noticed until 52 h;
4. On the 3rd day, the samples were further cured in an oven at 80°C for 12 h till no weight changes. The real dry film thicknesses were 40 ± 10 μm by the thickness gauge of the coating**.**

## 3 Aging test of natural weathering

As coatings were ready, the coated specimens were placed tightly in a fixed place under a natural outdoor environment for every 3 months of each season (spring, summer, winter and autumn) totally for 1 year. Data collection (temperature and humidity) of weather was recorded daily by the software weather version 11.01.524 of Xian city Beilin district. There is immense impact of different seasons on the coatings due to the varitions of the temperature and humidity.

### 3.1 Mechanical and chemical degradation of the coating

A property test was conducted gradually with aging to characterize mechanical and chemical properties degradation of coatings due to aging. As aging went a month, experiments analysis of peel-off, scratch, water contact angle, gloss measurements and electrochemical test were performed with three specimens. Simultaneously surface analysis was done by the optical microscope (VHX-5000) to observe the appearance of the coating. Perkin Elmer-Spectrum One conducted the FT-IR analysis on KBr disks in the region of 400–4,000 cm−1. The assessments for these tests are as follows.

### 3.2 Peel-off test

Peeling—off test is a method to quantify the adhesion of a coating to a substrate, which reflects the binding force required to remove the adhesive film from the substrate (Peng et al., 2016; Peng et al., 2017), Figure 2C.

The following formula is used to compute interfacial energy.

$$G=P\left(1-\cos\theta \right)/d-G_{\alpha }+G_{\beta }$$ (1)

*G*: interfacial energy, N/m.

*P*: critical force of film peeling to detach the film, N.

*D*: width of film at peeling point.

*Θ*: peeling angle.

*G*α: plastic bending dissipation force, N/m.

G_β_: participating strain force, N/m.

In the peeling-off test, there is no change in the length and width, so *G*_α_ = 0, hence, film thickness on the aluminum alloy surface is very thin so that it can be estimated as *G*_β_ = 0, so Eq. 2 becomes,

$$G=P\left(1-\cos\theta \right)/d$$ (2)

So, the evaluation result of G can be obtained from Eq. 2.

### 3.3 Scratch test

The Scratch test is a simple method to assess the anti-wear property. To conduct the scratch test, weighted sandpaper glides on the surface of a coated substrate at a certain speed to a certain distance, and the critical weight corresponding to a visible abrasive mark appearing was then detected for a unit width of sample Figure 2D.

### 3.4 Water contact angle test

Water drop contact angle measurements were done by a goniometer device to determine the liquid surface contact with each other. A drop of distilled water is deposited on a substrate surface with the help of a dropper at room temperature, and contact angles are measured by a micro charged-coupled device (CCD) camera for respective images.

### 3.5 Electrochemical test

PARSTAT 2273 (Princeton Applied Research Inc.) performs EIS (electrochemical impedance spectroscopy) and potentiostatic polarization. A three-electrode system was used to reduce and compensate for the potential changes caused by large currents passing through the working (Al alloy 6101) electrode, saturated calomel electrode as a reference, and 0.1 cm^2^ Pt (platinum) as a counter electrode.

### 3.6 Surface characterization

The surface of the coated Al alloy 6101 specimens was also analyzed by the SEM and EDS (Uanta FEG 250) characterization to confirm the complete mixing, dispersion, elemental, and phase identification of the pigments in the epoxy coatings. Moreover, the specimens’ optical images were also taken after the three-month aging period, so the final surface of the neat and ZP modified epoxy coatings were analyzed.

### 3.7 Gloss evaluation

The perception of the gloss can relate to a product (substate) finish, texture and how the sample is illuminated and viewed. Surface with high reflectance and perceived as a gloss, shiny or lustrous, while the less reflective surface is perceived as a semigloss or matt (Calvez et al., 2022). Nevertheless, in many instances, a high gloss finish implies high quality, and it is important to ensure that the gloss level not only remains consistent across the substrate, which may negatively affect the performance of the metal substrate. The most common angle of 60° is used to measure the gloss of the coated surface of the Al alloy 6101 after the preparation of the samples; the gloss meter WG60 is used to measure the readings bypassing the LED light from the gloss meter at the fixed angle through the sample (Wernståhl, 1996) Figure 2E. While the following formula measured the gloss retention (%)

$$Gloss=\frac{R_{s\left(t\right)}}{R_{STD}}\times 100$$ (3)

Rs(t) represents the specular resistance divided by the highly polished reflectance, black glass standard RSTD and multiplied by 100. The calibration of the gloss meter was done by the very highly polished black surface having a refractive index of 1.611 for the D sodium line assigning the value of 100 with all geometries. However, the initial surface brightness of the substrate was measured after sample preparation and the (%) change in gloss measurements before and after the aging test is known as gloss retention. Gloss retention was calculated by the following formula (Hannu et al., 2017; Korhonen et al., 2018).

$$Gloss\;retention=\frac{Gloss\;aberrated}{Gloss\;initial}\times 100\%$$ (4)

## 4 Result and discussion

### 4.1 FTIR analysis

**FIGURE 3:**
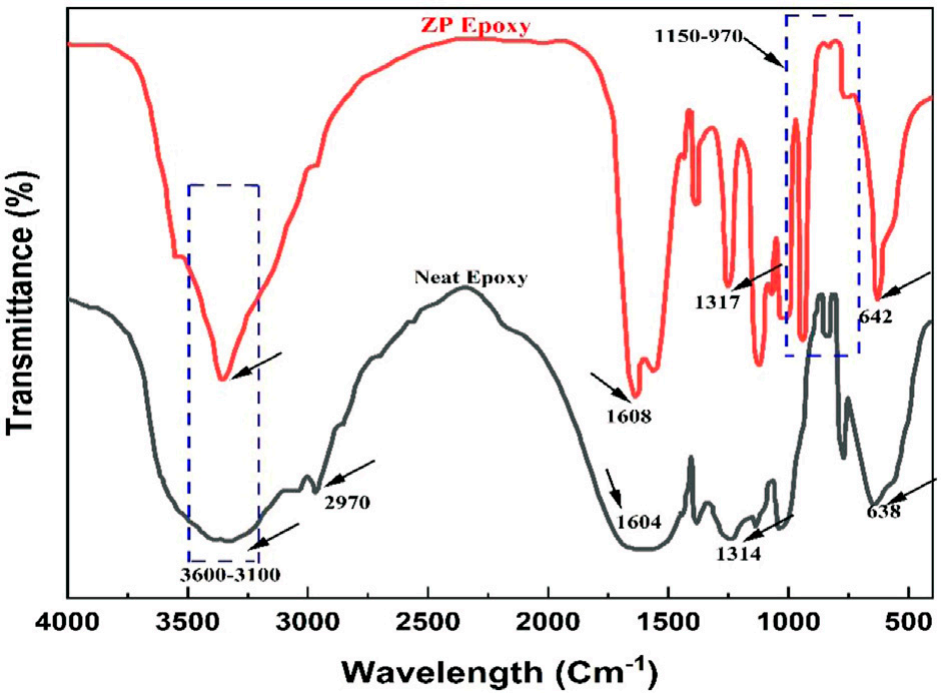
FT-IR of neat and modified epoxy coating surfactant.

Figure 3 presents the spectral FT-IR of neat and ZP-modified epoxy coatings through different surfactants. Wide-ranging 3,600–3,100 cm^-1^ absorption bands demonstrate vibration stretching of H-O adsorption peaks (Yan et al., 2009). Absorption bands present between 1,630 and 1,610 cm^-1^ parallel to H-O-H absorption vibration peaks, demonstrating that water crystals are present in samples. At 1,150–970 and 645–635 cm-^1,^ absorption bands are attributed to bending and stretching vibration peaks of the PO_4_^3-^ group (Yuan et al., 2006; Zhou et al., 2015). However, these analyses conclude that pigment mixing on the substrate is obvious and successfully deposited during the experimental stage.

### 4.2 SEM and EDS

**FIGURE 4:**
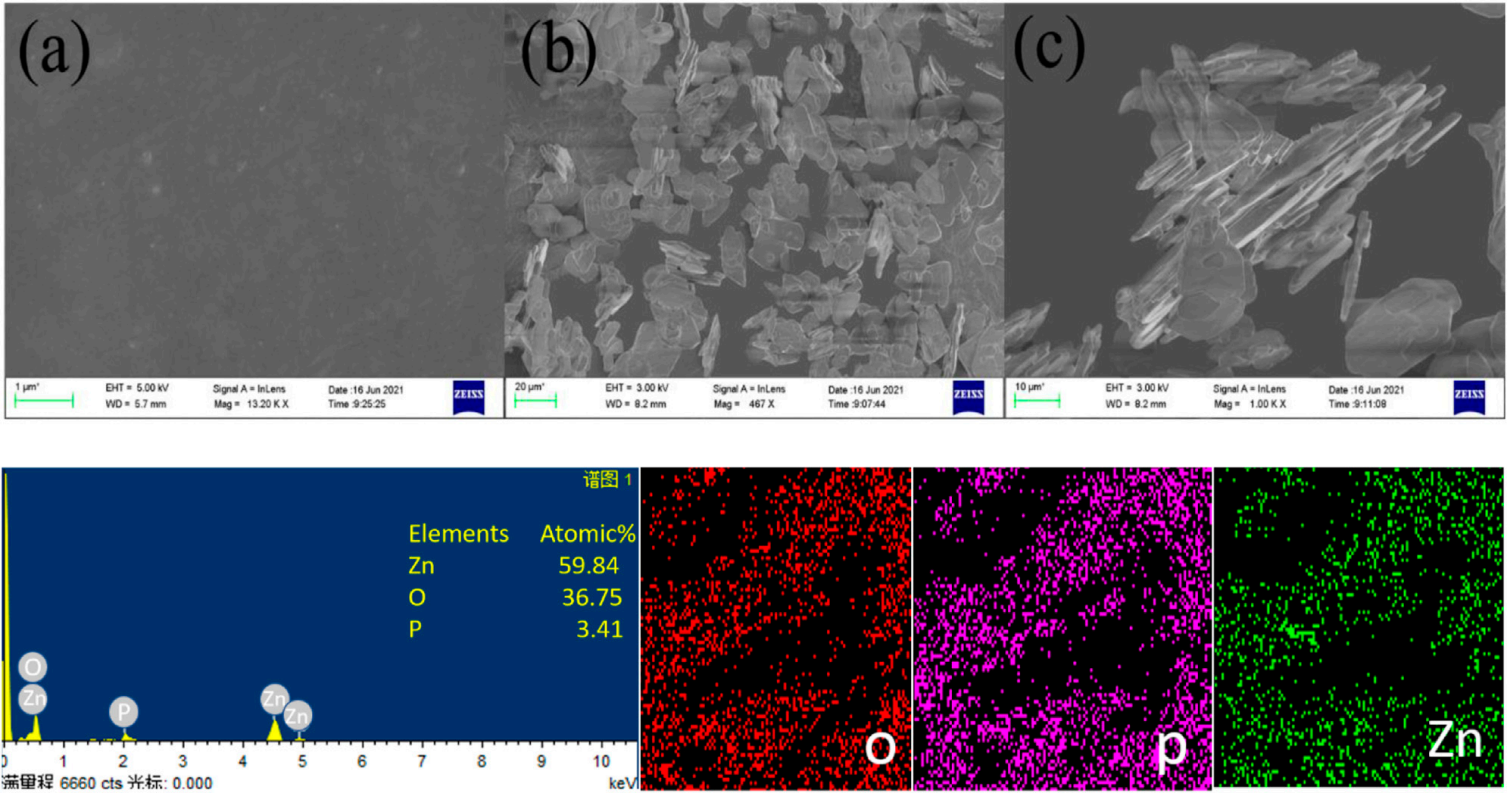
SEM images of ZP modified epoxy coating **(a–c)** showing complete dispersion and microstructures of pigments without agglomeration while EDS shows elemental analysis.

The epoxy coating has varying ZP pigments, and the scanning electron microscope demonstrated that the correct dispersal of the pigment through EDS analysis in the solution suggests. For preparing the epoxy solution with ZP pigments, the calculated quantity of ZP pigments in different concentrations has given the best results for the adherence test since adding a calculated amount of anti-corrosion pigments to the epoxy solution can affect the chemical structure and the content of pigments with force (Yuan et al., 2006; Zhou et al., 2015). Thus, the binding force and corrosion protection, according to the literature, are two key feasible causes for improving the anti-corrosion pigment coatings. The first is the complete diffusion of the pigments to boost adherence characteristics in the epoxy solution Figure 4 (a) showing complete smooth surface after application of pigmented coating while Figures 4B, C are the indication of microsize of pigments and no formation of agglomertaion. Another explanation might be creating the shielding layer on the substrate in the presence of electrochemically active pigments. In addition, data from EDS (Figure 4) revealed the elements on the covered surface by an interaction between the electron beams revealing various phosphate (P) and zinc (Zn) atomic spectrums (Ramezanzadeh et al., 2022; Yi et al., 2022). The mapping also showed that the ZP pigments in the epoxy solution were equally and properly dispersed. The presence of Zinc and phosphorus ions on Al alloy 6101 shows that it can protect metals against corrosion.

### 4.3 Aging effect in results of peel-off and scratch test

**FIGURE 5:**
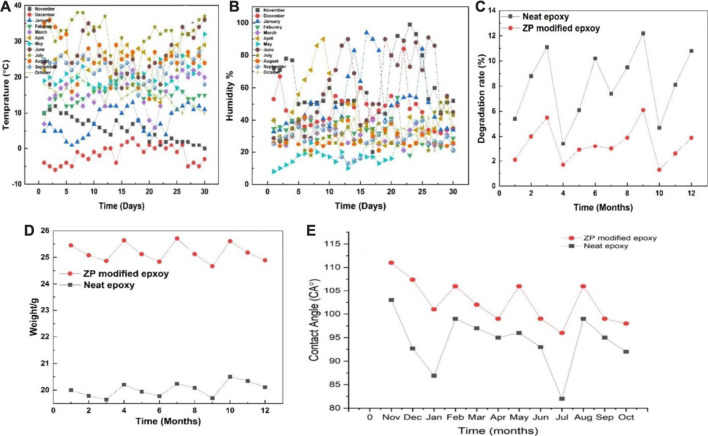
**(A, B)** Changes of temperature and humidity data in Xi’an, China, 5 **(C, D)** Degradation of coating of neat epoxy and ZP pigments modified epoxy peeling-off force and scratch visible weight, while 5**(E)** showing contact angles of the neat and ZP modified coating’s degradation throughout the year.

Coated Al alloy 6101 suffered the natural aging of Xi’an China from November 2020 to October 2021. Figures 5A, B show the changes in humidity and temperature data in Xi’an, China. The maximum humidity recorded is 80%–90% during the rainy day, while the maximum temperature was 35°C–38°C. Table 2 shows the results of the peeling-off force of the coating on Al alloy 6101 vs. aging at 90° angle for epoxy coatings and ZP pigments modified epoxy coatings; Table 3 shows the results of critical load on coated Al alloy 6101 for epoxy coatings and ZP pigments modified epoxy coatings; Figures 5C, D show the degradation rates of peel-off force and scratch visible weight, respectively. It can be seen from Tables 2, 3 that ZP pigments modified epoxy coatings have strong resistance against physical aging.

**TABLE 2:** Peeling-off force of the coating on Al alloy 6101 with ZP modified and near epoxy aging at 90° angle.

Samples	θ/°	d/m	Neat epoxy			ZP modified epoxy		
Time (months)			F/N	G/N.m^ˉ1^	Degradation rate	F/N	G/N.mˉ^1^	Degradation rate
Without aging	90	9.7 × 10^−3^	0.144	14.7	—	0.223	22.8	—
November	90	9.7 × 10^−3^	0.133	13.6	5.4%	0.213	21.9	2.1%
December	90	9.7 × 10^−3^	0.130	13.3	8.8%	0.142	22.4	4%
January	90	9.7 × 10^−3^	0.126	12.9	11.1%	0.215	22.0	5.5%
February	90	9.7 × 10^−3^	0.164	14	3.4%	0.209	21.4	1.7%
March	90	9.7 × 10^−3^	0.131	13.5	6.1%	0.221	22.4	2.9%
April	90	9.7 × 10^−3^	0.128	13.1	10.2%	0.213	21.9	3.2%
May	90	9.7 × 10^−3^	0.136	13.9	7.4%	0.207	21.6	3.0%
June	90	9.7 × 10^−3^	0.131	13.4	9.5%	0.216	22.1	3.9%
July	90	9.7 × 10^−3^	0.126	12.9	12.2%	0.214	21.9	6.1%
August	90	9.7 × 10^−3^	0.139	14.2	4.7%	0.209	21.4	1.3%
September	90	9.7 × 10^−3^	0.135	13.8	8.1%	0.221	22.5	2.6%
October	90	9.7 × 10^−3^	0.136	13.2	10.8%	0.217	22.2	3.9%

**TABLE 3:** Comparison of critical load on coated Al alloy 6101 for ZP modified and neat epoxy aging.

Samples	Speed mm/min	Scratch visibility	Neat epoxy weight/g	ZP modified epoxy weight/g
Without aging	0.04	Visible	20.98	26.37
November	—	—	20.00	25.45
December	—	—	19.79	25.08
January	—	—	19.65	24.87
February	—	—	20.21	25.64
March	—	—	19.94	25.12
April	—	—	19.78	24.84
May	—	—	20.24	25.71
June	—	—	20.09	25.12
July	—	—	19.70	24.67
August	—	—	20.50	25.61
September	—	—	20.35	25.18
October	—	—	20.11	24.89

The peeling force of ZP pigments modified epoxy coatings is 50% higher than that of the neat epoxy coatings. Better protection and adhesion are obtained from ZP pigments-modified epoxy coating, which mainly depends upon the complete diffusion and mixing of ZP pigments in the coating compared to the neat epoxy.

### 4.4 Contact angle test of coating

As to a contact angle test, if the contact angle is greater than 90°, the surface is hydrophobic; otherwise, it is hydrophilic. The test results of contact angles of bare and coated Al alloy 6101. The contact angles for bare Al alloy 6101, epoxy coating and ZP pigments modified epoxy coating is 86.1°, 106°, and 108.6°, respectively, which shows a significant improvement of hydrophobic property of the surface by ZP addition. Moreover, a decreasing trend for seasonal variations was also observed. Figure 5E represents the contact angle variations throughout the year according to the surface degradation of the neat epoxy and ZP-modified epoxy. Concerning time, the coated samples’ surface became rougher and degraded due to the physical aging phenomena, and the surface became more hydrophilic.

### 4.5 Electrochemical results

#### 4.5.1 Potentiodynamic polarization measurement (PDP)

**FIGURE 6:**
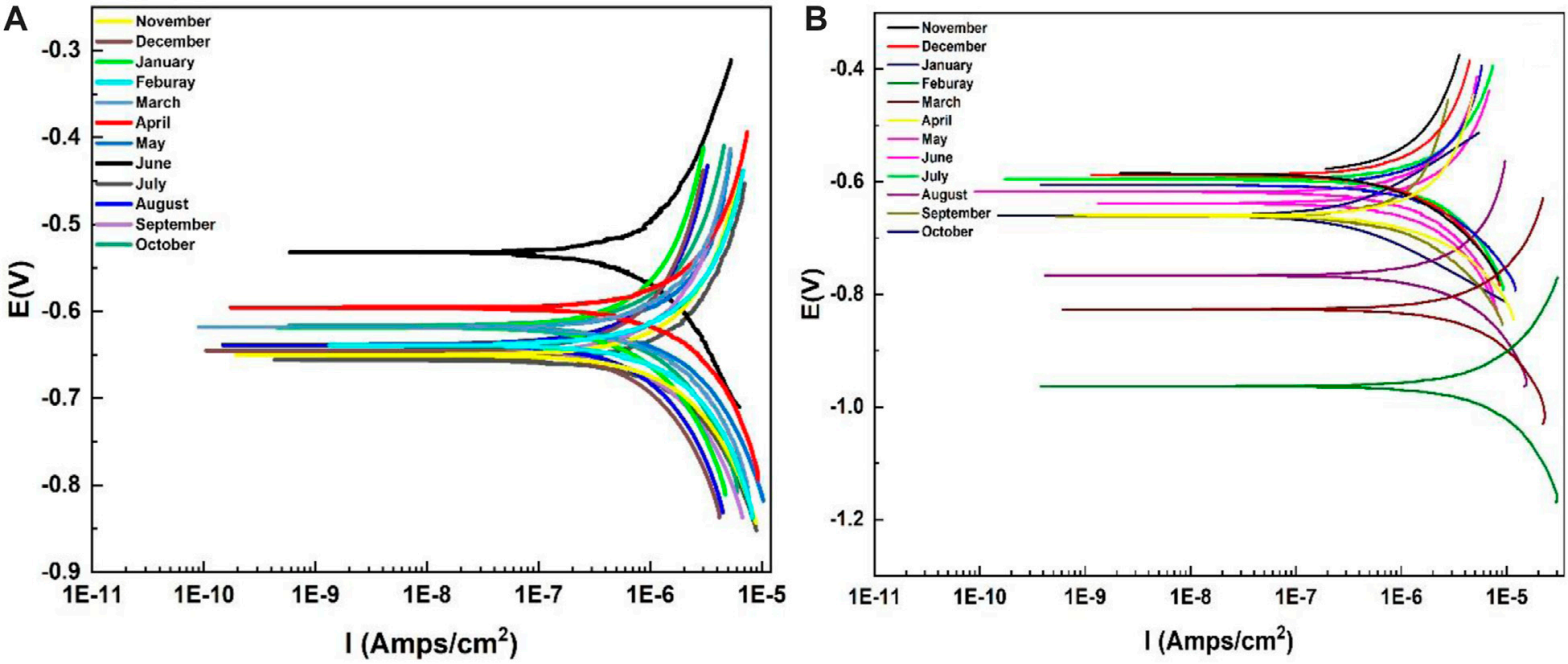
Polarization curves of epoxy in the alkaline solution of NaOH with pH 11 for 1-year **(A)** ZP pigments mod. Epoxy; **(B)** Neat epoxy.

Polarization curves obtained for Al-6101 in the alkaline media with pH 11 deviations are shown in Figure 6. Parameters of the polarization curves are also given in Table 4, which includes the corrosion current density (i_corr_), corrosion potential (E_corr_), Tafel slopes (βc, βα), and annual corrosion rate (mm/a) by extrapolating cathodic and anodic polarization curves to E_corr_. In both cases, the decreasing trend of the corrosion potential of the coated surface of Al-6101 is shifting towards negative values (Zubielewicz and Krolikowska, 2009; Zhou et al., 2022). In addition, this decreasing trend is more observed in neat epoxy coatings. It can be observed that the corrosion rate is approximately four times higher in the aging specimens for summer months, which is a remarkable reduction in the i_corr_ of ZP pigment-modified samples in the presence of an alkaline solution with pH 11. Similarly, the corrosion protection efficiency of ZP-modified samples is very high, 0.070 mm/a while 0.198 mm/a for neat epoxy, which is much lower after the summer aging experiments. The corrosion rate of ZP pigments modified epoxy coatings is about 70% lower than that of neat epoxy coating (Zheng et al., 2022).

**TABLE 4:** Electrochemical measurements for ZP modified and neat epoxy coating on Al alloy 6101 samples in corrosion solution.

		Neat epoxy					ZP modified epoxy				
Samples	pH	E_0_/V	I_0_/Amp/cm^2^	Ba/mV	Bc/mV	Cr (mm/a)	E_0_/V	I_0_/Amp/cm^2^	Ba/mV	Bc/mV	Cr (mm/a)
Without aging	11	−0.69	1.155 × 10^−5^	49.25	215.7	0.009	−0.66	1.023 × 10^−5^	23.82	149.5	0.003
November	—	−0.64	2.091 × 10^−5^	143.2	122.3	0.042	−0.61	2.091 × 10^−5^	145.34	136.2	0.011
December	—	−0.54	2.924 × 10^−5^	202.6	242.2	0.087	−0.59	2.924 × 10^−5^	187.24	142.3	0.018
January	—	−0.61	9.011 × 10^−6^	206.2	263.4	0.112	−0.63	9.011 × 10^−6^	136.26	156.3	0.029
February	—	−0.63	2.141 × 10^−5^	147.3	112.7	0.005	−0.67	2.091 × 10^−5^	147.25	104.3	0.015
March	—	−0.57	2.741 × 10^−5^	187.5	145.3	0.089	−0.59	2.924 × 10^−5^	123.15	113.4	0.023
April	—	−0.68	9.168 × 10^−6^	169.8	163.2	0.124	−0.63	9.011 × 10^−6^	165.22	178.3	0.036
May	—	−0.63	2.909 × 10^−6^	130.7	102.9	0.077	−0.61	2.091 × 10^−5^	102.67	196.6	0.020
June	—	−0.82	3.321 × 10^−5^	162.4	149.1	0.172	−0.59	2.924 × 10^−5^	145.28	121.2	0.040
July	—	−0.96	8.195 × 10^−5^	189.1	221.2	0.198	−0.65	9.011 × 10^−6^	190.66	136.3	0.070
August	—	−0.66	2.741 × 10^−5^	122.9	141.1	0.041	−0.62	2.091 × 10^−5^	189.32	141.3	0.015
September	—	−0.54	2.963 × 10^−5^	119.2	243.2	0.083	−0.55	2.924 × 10^−5^	174.81	124.2	0.021
October	—	−0.51	9.751 × 10^−6^	185.3	323.4	0.132	−0.66	9.011 × 10^−6^	114.12	163.4	0.034

#### 4.5.2 Electrochemical impedance spectroscopy

**FIGURE 7:**
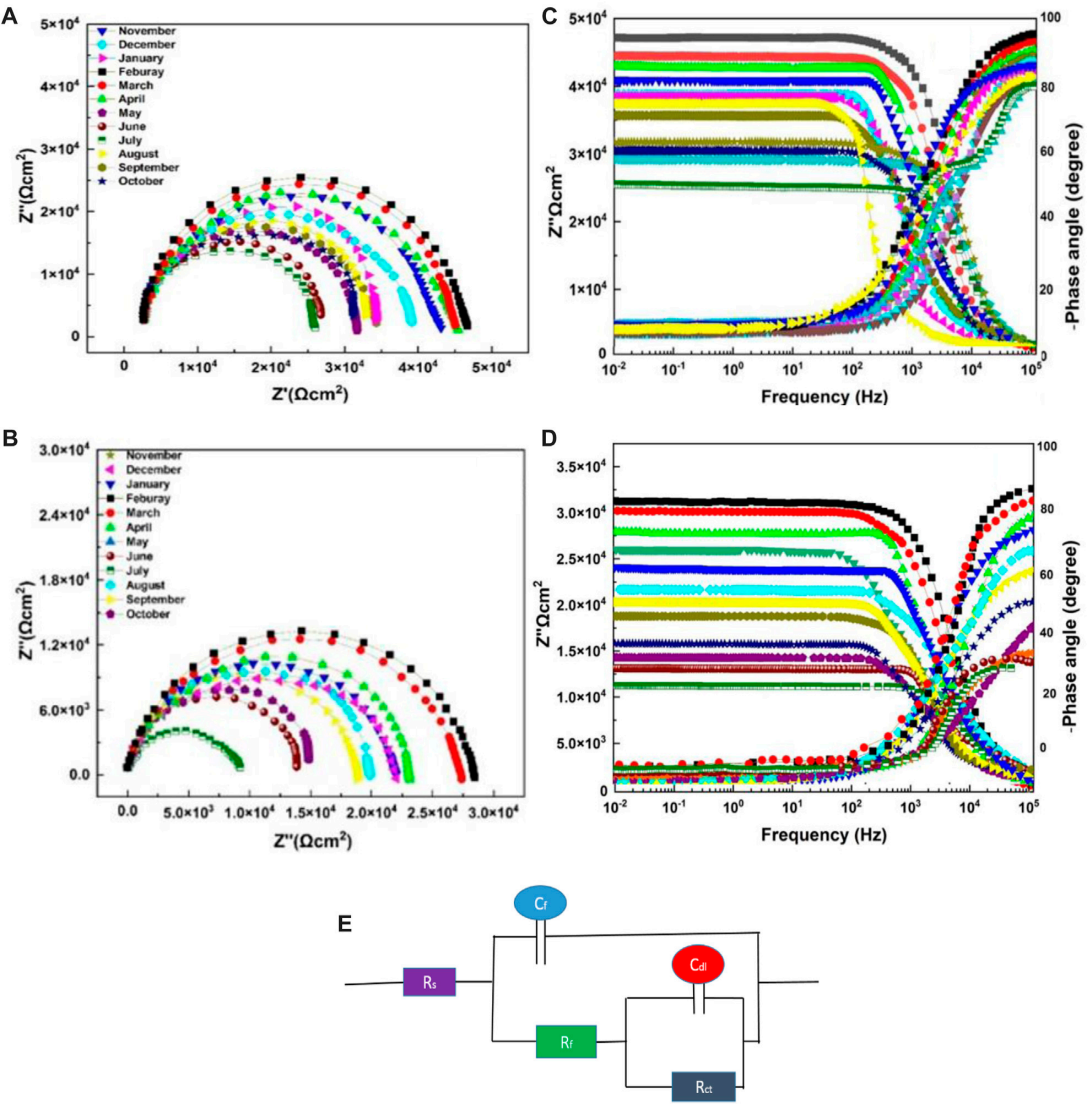
Nyquist plot and Bode plots for EIS analysis in an alkaline solution of NaOH with pH 11 **(A)** and **(C)** with ZP Pigment; **(B)** and **(D)** neat epoxy: **(E)** EEC model.

The most common and typically useful technique to test the corrosion events occurs on the surface of the working electrode, where pH becomes highly basic at cathodic and acidic at anodic sites. EIS measurements were conducted to evaluate the corrosion protection performance of the neat epoxy coatings with the modified ZP pigments. This analysis is performed in an alkaline solution of NaOH with pH 11 for all specimens. Figures 7A, B are the Nyquist plots showing the semicircle arc of the samples with different aging months, and the decreasing trend can be seen in the figure due to the degradation of the coatings. Moreover, the gradual decrease of Rct from 7.76 × 10^5^ to 1.69 × 10^4^, and 7.76 × 10^5^ to 1.63 × 10^4^ in summer months aging. Table 5 shows electrochemical parameters from the EIS equivalent electrical circuit of specimens. The electric resistance of ZP pigments modified organic–silicone epoxy coatings is about 30% higher than that of neat organic–silicone epoxy coatings. ZP pigment-modified coatings have better properties due to ZP appearing in the coating, which effectively resists the physical and mechanical degradation of the coatings (Khan et al., 2022d).

**TABLE 5:** Electrochemical parameters from EIS equivalent electrical circuit of ZP pigment-modified epoxy specimens.

Samples		Neat epoxy				ZP modified epoxy			
Time (months)	Rs (Ω⋅cm^2^)	Cf (S⋅sn/cm^2^) (n)	Rf (Ω⋅cm^2^)	Cdl (S⋅sn/cm^2^) (n)	Rct (Ω⋅cm^2^)	Cf (S⋅sn/cm^2^) (n)	Rf (Ω⋅cm^2^)	Cdl (S⋅sn/cm^2^) (n)	Rct (Ω⋅cm^2^)
Without aging	0.1	1.353×10^−8^	3.566×10^4^	2.14×10^−8^ (0.84)	6.91×10^4^	1.353×10^−9^	3.566×10^4^	1.28×10^−5^ (0.95)	5.36×10^5^
November	—	2.362×10^−8^	4.146×10^4^	5.52×10^−8^ (0.80)	7.32×10^4^	1.623×10^−8^	2.315×10^5^	3.24×10^−5^ (0.95)	4.36×10^5^
December	—	2.352×10^−8^	4.963×10^4^	6.14×10^−8^ (0.96)	5.32×10^4^	2.585×10^−8^	1.873×10^5^	3.47×10^−5^ (0.81)	5.32×10^4^
January	—	3.654×10^−8^	4.871×10^4^	5.47×10^−8^ (0.91)	4.39×10^3^	2.082×10^−8^	1.845×10^4^	4.23×10^−6^ (0.85)	7.69×10^5^
February	—	2.365×10^−8^	4.321×10^4^	4.74×10^−8^ (0.96)	6.32×10^4^	4.161×10^−6^	2.033×10^5^	4.98×10^−6^ (0.84)	8.71×10^5^
March	—	2.847×10^−8^	5.351×10^4^	5.85×10^−8^ (0.94)	6.12×10^4^	7.767×10^−6^	4.161×105	4.46×10^−6^ (0.90)	6.11×10^4^
April	—	3.621×10^−8^	6.354×10^4^	5.32×10^−8^ (0.85)	5.32×10^3^	3.475×10^−6^	2.214×10^4^	4.25×10^−6^ (0.81)	4.05×10^5^
May	—	2.059×10^−8^	4.247×10^4^	4.22×10^−8^ (0.87)	2.62×10^4^	9.196×10^−7^	1.354×10^5^	2.14×10^−5^ (0.84)	7.76×10^5^
June	—	3.649×10^−8^	5.385×10^4^	5.55×10^−8^ (0.82)	2.59×10^3^	2.088×10^−6^	1.963×10^6^	1.53×10^−6^ (0.91)	4.12×10^4^
July	—	3.545×10^−8^	7.147×10^4^	4.76×10^−8^ (0.88)	1.42×10^2^	7.804×10^−6^	1.364×10^5^	2.65×10^−8^ (0.83)	2.28×10^3^
August	—	2.365×10^−8^	5.321×10^4^	4.22×10^−8^ (0.94)	5.74×10^4^	3.354×10^−7^	5.324×10^6^	4.48×10^−6^ (0.89)	4.32×10^5^
September	—	3.214×10^−8^	4.825×10^4^	4.22×10^−8^ (0.81)	4.87×10^4^	2.024×10^−6^	2.124×10^6^	1.66×10^−5^ (0.88)	6.32×10^4^
October	—	2.365×10^−8^	4.135×10^4^	4.22×10^−8^ (0.85)	4.12×10^3^	4.532×10^−6^	3.214×10^5^	7.18×10^−6^ (0.81)	1.32×10^4^

### 4.6 Gloss measurement analysis

Gloss measurements conclude that surface degradation and deprivation due to environmental factors progressively decrease. The shine or gloss of the coated samples after the coating and after the physical aging was very different; gloss was just like removed from the samples, and surfaces seemed to be more rough, uneven and dull. A comparison can be seen in Table 6 that the gloss retention in the neat and modified epoxy is very obvious. The gloss removal from the neat epoxy is very fast and high compared to modified epoxy. Reasons could be the evaporation rate of the neat epoxy being high under the natural environment and UV radiation, spacing present between the molecules of the neat epoxy is high. At the same time, those spaces were covered by adding the Zp pigment in the epoxy during the modification, which keeps the epoxy intact to the substrate surface and enhances the efficiency of the coatings. It is worth noting that the gloss retention is 51.34% during the summer season in the neat epoxy. However, the ZP-modified epoxy gloss retention was 69.10%. Moreover, it is proof that after modification to the epoxy, the surface of the experimental metal can be protected more, and its durability, including the esthetics of the surface (metals), can also be maintained well for a long period under harsh weather conditions.

**TABLE 6:** Mean and standard deviation values of the gloss retention of both neat and ZP-modified epoxy coatings.

Aging months	Neat epoxy		ZP modified epoxy	
Before aging	Gloss GU 198.01	GR% 100	Gloss GU 198.01	GR%
November	181.26 ± 1.36	91.54	187.14 ± 1.41	94.51
December	169.32 ± 2.58	85.51	176.62 ± 2.48	89.20
January	147.71 ± 3.41	74.60	159.37 ± 3.51	80.48
Feb	186.21 ± 1.11	94.10	190.74 ± 1.36	96.33
March	171.87 ± 2.32	86.90	182.36 ± 2.47	92.10
April	151.32 ± 3.25	75.52	168.85 ± 3.65	85.27
May	173.58 ± 1.21	87.74	182.21 ± 1.01	91.91
June	152.25 ± 2.21	76.91	169.45 ± 2.36	85.58
July	101.52 ± 3.34	51.34	136.74 ± 3.75	69.10
August	189.35 ± 1.31	95.55	193.98 ± 1.29	97.96
September	166.67 ± 2.47	84.17	174.35 ± 2.51	88.02
October	141.12 ± 3.61	71.27	156.52 ± 3.49	79.05

### 4.7 Optical microscopy of coatings

**FIGURE 8:**
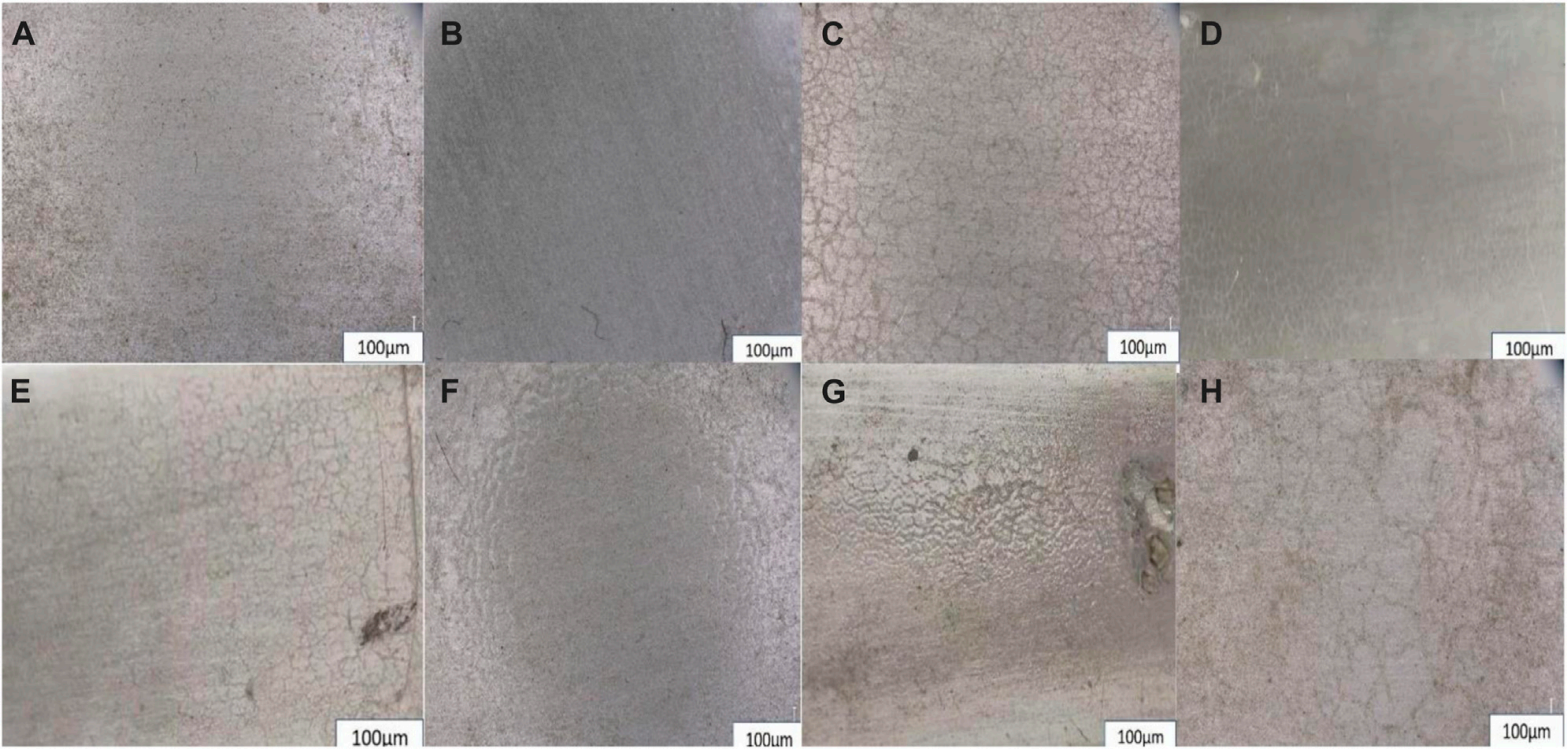
ZP modified epoxy coating throughout the year **(A)**January, **(B)**April, **(C)**July and **(D)** October optical images of coatings and neat epoxy coating of throughout a year **(E)**January, **(F)**April, **(G)**July and **(H)** October optical images of coatings.

Figure 8 shows the surface appearance of the aged samples observed using an optical microscope. As the time of physical aging is increasing, the protective efficiency of the epoxy coating decreases. Additionally, it can be seen clearly that the neat epoxy is losing its gloss, and cracks are more visible on the surface of the epoxy coatings than on the ZP-modified epoxy coatings. The appearance and protective durability of ZP pigmented specimens’ modified coatings are much extraordinary though cracks and shrinkage can also be observed due to the shielding effect of ZP pigments. The modified surface appearances are still much better compared to neat epoxy after the final optical images taken after the three months of experimentation during the one season. The following images show that the cracks and scissures are more prominent in the neat epoxy coating during the summer season; in both coatings, the neat epoxy seems more degraded, rough and bumpy surface. On the other hand, the ZP-modified epoxy coating surface still looks much better and smooth, although some cracks and dust appear on the substrate surface.

## 5 Conclusion

Through the comparative study of neat epoxy and ZP pigment-modified epoxy coatings on Al alloy 6101 under natural weathering conditions after aging in Xi’an, China, for 12 months, the following conclusion is drawn.

1. Peeling force of ZP pigments-modified epoxy coatings is 50% higher than that of the neat epoxy coatings though both peel-off adhesion strength and scratch test visibility decreased in both coatings
2. Totally, the electric resistance of ZP pigments-modified epoxy coatings is about 30% higher than that of neat epoxy coatings, and the corrosion rate of ZP pigments-modified epoxy coatings is about 70% lower than that of neat epoxy coatings.
3. Optical surface observation of the coatings showed that the ZP-modified epoxy coating could effectively restrict the crack and shrinkage in coatings after aging.
4. Gloss measurements and surface analysis also proved that the neat epoxy coating lost 50% of its gloss, and the surface became rougher after aging compared to the ZP-modified epoxy coating.
5. Maximum degradation and surface deterioration occur in the summer compared to other seasons; the main reason could be the Influence of the maximum UV radiation, temperature and high humidity in the atmosphere.

## Data Availability

The original contributions presented in the study are included in the article/supplementary material, further inquiries can be directed to the corresponding authors.
